# β-adrenergic receptors modulate CA1 population coding and synaptic plasticity during cumulative spatial memory formation and updating

**DOI:** 10.1038/s41598-026-40218-x

**Published:** 2026-02-19

**Authors:** Ninad Shendye, Josué Haubrich, Jens P. Weber, Hardy Hagena, Denise Manahan-Vaughan

**Affiliations:** Medical Faculty, Department of Neurophysiology, Ruhr University Bochum, Universitätsstr. 150, MA 4/150, 44780 Bochum, Germany

**Keywords:** Mouse, *In vivo* calcium imaging, Learning and memory, Hippocampus, Neuronal ensemble, Synaptic plasticity, Adrenergic, Neuroscience, Physiology

## Abstract

Hippocampal neuronal ensembles are likely to support the acquisition, stabilization and updating of spatial experience. Spatial learning is typically cumulative, but little is known about how neuronal ensembles are manifested during this process. Here, we used wide-field Ca^2+^-imaging and in vivo electrophysiology to monitor CA1 pyramidal cells and synapses, respectively, during cumulative item-place learning in adult male CBA/CaOlaHsd mice. In controls, we observed refinement in neuronal activation patterns along with reduced reactivation in response to novel item-place configuration. Synaptic plasticity responses, place cell-like properties, population burst activity, and functional connectivity were consistent with the encoding and updating of item-place memory. Noradrenaline activity via β-adrenergic receptors (β-AR) is a critical modulator of CA1 function, but its role in regulating cellular dynamics during cumulative item-place learning remains unclear. To examine this, we pharmacologically antagonized β-AR prior to the 1st item-place exposure. This led to reduced cellular recruitment, disrupted ensemble reactivation, impaired synaptic plasticity, reduced spatial tuning, dampened population bursts, and altered functional connectivity within neurons. This was accompanied by impaired spatial learning compared to controls. Our results reveal the population activity of CA1 neurons during item-place learning and show that β-AR support memory function by influencing both neuronal and network-level dynamics.

**Supplementary Information:**

The online version contains supplementary material available at 10.1038/s41598-026-40218-x.

## Introduction

The formation and updating of episodic-like memories rely on the hippocampus, particularly the CA1 region, which integrates spatial and contextual information to support flexible memory representations^1^. The interplay between cellular activity and population-level synchrony within CA1 is essential for encoding new experiences and incorporating them into existing memory frameworks^2–4^. Item-place associations, linking object identities to their spatial locations, are core components of “what-where” information in episodic memories^5^. Across repeated episodes, such associations can stabilize, or update, memories for experienced events^6^. Although noradrenaline (NA) acting on β-adrenergic receptors (β-AR) is known to modulate memory in CA1^7^, its specific role in shaping cumulative item-place learning remains unresolved.

Neuronal ensembles activate in a coordinated manner during memory encoding^8^, and their reactivation supports memory recall^4,9,10^. The hippocampus is central to these processes, mediating memory encoding, consolidation, recall, and updating^11–13^. Moreover, the hippocampus is known to be involved in episodic memory processing that relies on the ability to learn, store, and retrieve information about unique personal experiences^14^. Within the hippocampus, CA1 neurons encode spatial contexts to create cognitive representations of space^2^. During memory encoding and consolidation, CA1 neurons exhibit periodic bursts of synchronous activity^15–17^ and the reactivation of neurons that participated in these bursts during information encoding has been linked to memory updating^18^. Although electrophysiological recordings from place cells^19^ as well as loss and gain-of-function studies of CA1 pyramidal cells^20^ have demonstrated the necessity of population activity for the mapping of space, as well as for the acquisition, retrieval and updating of spatial memories, little is known about how population dynamics in terms of ensemble activity and neuronal connectivity are engaged during cumulative spatial learning.

Multi-unit electrophysiological recordings from neurons during associative learning have revealed transregional co-activity of neurons in cortex and subcortex^21^, whereas recordings from place cells have revealed how context and motivation influences place fields^19,22,23^. However, not all CA1 pyramidal cells are place cells^24^ and neuronal population dynamics can reflect a multitude of other forms of information processing such as stimulus-dependent sensory integration^25^ behavioral/affective state^26^ and temporal experience^27^. Observing the activity of pyramidal cells during cumulative learning and/or information updating can thus, provide novel insights into functional segregation within the CA1 region and demonstrate how neuronal ensembles are modified by experience^28^. Novel approaches, such as wide-field Ca^2^-imaging of hundreds of CA1 neurons offer an approach through which neuronal ensembles can be monitored in real-time in behaving rodents^29^.

Synaptic plasticity enables the encoding of information by altering the strength of synaptic transmission between neurons^30^ and is modulated by neuromodulators such as NA. The NA system uniquely modulates hippocampal function by improving neuronal responses towards discrete stimuli through enhanced signal-to-noise ratios, facilitating information detection and encoding^31–33^, and it more broadly plays a critical role in arousal and vigilance^30^. Importantly, NA acting on β-AR is a key determinant of whether new experiences result in persistent hippocampal synaptic plasticity^7,34–36^. β-AR are densely expressed in the hippocampus^7^ and play a central role in promoting synaptic plasticity in the CA1 region^5,6,37–39^. The importance of β-AR for spatial learning and memory has been demonstrated across diverse rodent tasks. Moreover, noradrenergic neuromodulation can alter the location preference of place cells^40^ and neuronal excitation in the hippocampus^34^.

In this study, we leveraged wide-field Ca^2+^-imaging of neurons of the dorsal hippocampus CA1 region to investigate how neuronal ensembles develop, stabilize and/or change, during item-place learning and the updating of item-place information in mice. We also used in vivo electrophysiological recordings from CA1 synapses to explore the extent to which synaptic plasticity emerges during the sequence of behavioral events implemented in the study. To assess the role of β-AR in these processes, we examined how acute pharmacological antagonism of β-AR, during memory encoding, affects subsequent recall and updating of item-place associations, as well as the associated changes in neuronal ensemble behavior and synaptic plasticity. Our findings reveal the engagement of CA1 neurons, during information acquisition and updating item-place information, at multiple levels including neuronal recruitment, population activity, functional connectivity and place-field mapping. Hippocampal synaptic plasticity in the form of long-term depression (LTD) emerged during novel memory acquisition and information updating, but not during reiteration of exposure to the same spatial experience. These patterns of activity reflect information acquisition, stabilization and updating. Moreover, we show that β-adrenergic antagonism impaired item-place memory and that this disruption is accompanied by changes in hippocampal neuronal activity, alteration of neuronal ensemble profiles, and impairments of synaptic plasticity.

## Results

### Pharmacological Inhibition of β-adrenergic receptors impairs item-place learning

For this study, we used a cumulative item-place learning and information updating task that is known to induce hippocampal synaptic plasticity and is regulated by β-AR^6^. We monitored both behavioral performance and neuronal Ca^2+^ activity when mice were first exposed to a novel item-place configuration, were re-exposed to the same configuration 1 h later, and then experienced a change of item-place configuration after a further 60 min. To examine the role of β-AR in these learning events, we administered the antagonist propranolol systemically, before task initiation.

**Fig. 1:**
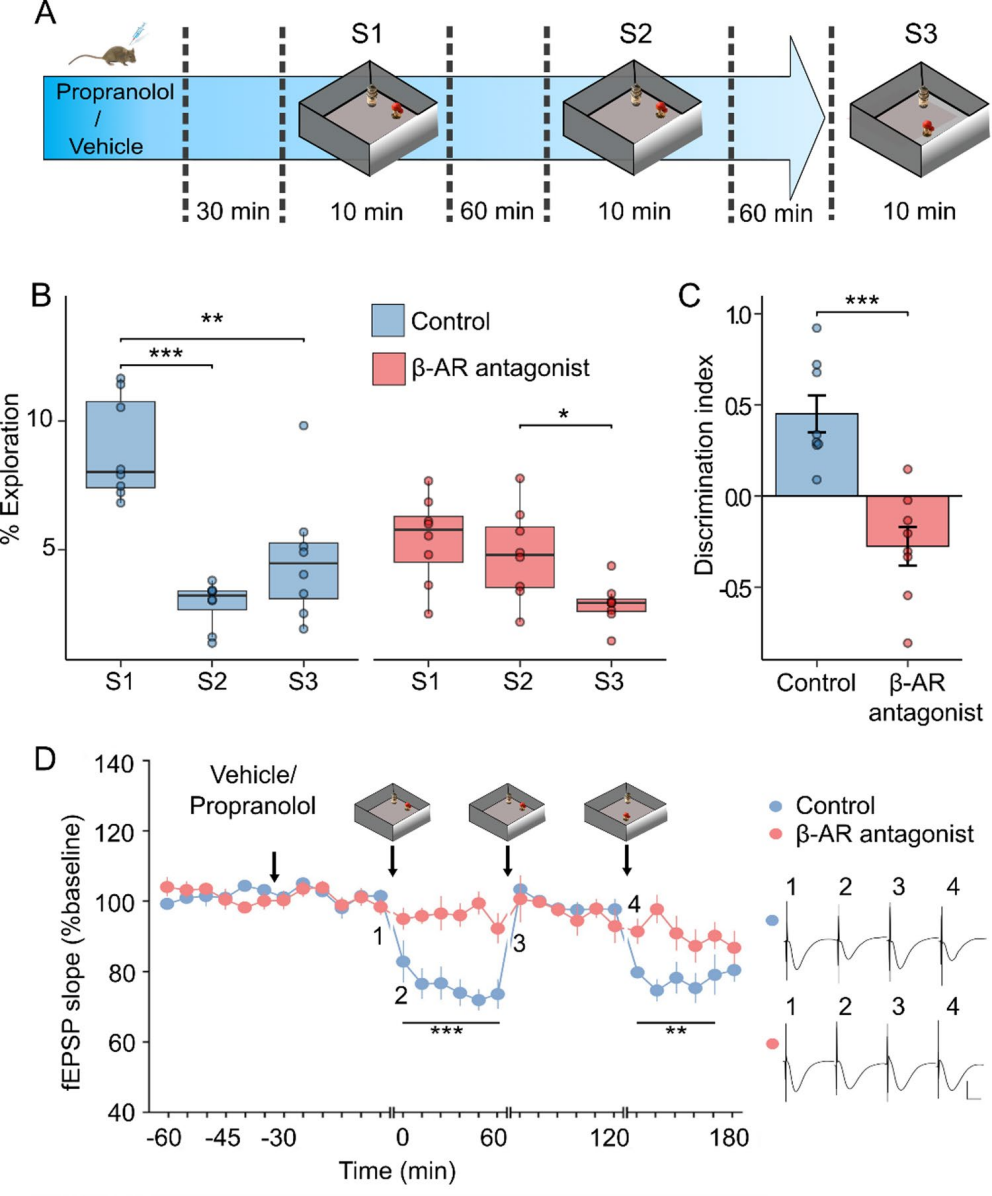
β-AR antagonism impairs the encoding of item-place associations and impairs learning-facilitated synaptic plasticity. **A**) Schematic representation of the experimental design for the item-place learning task consisting of session 1 (**S1**, novel item-place configuration); session 2 (**S2**, re-exposure to the same item-place configuration) and, session 3 (**S3**, displacement of one of the now familiar objects to a different location). Sessions were 10 min long and spaced by 60 min. Vehicle or propranolol-treatment occurred 30 min before session 1. **B**) Object exploration times expressed as a percentage of total arena exploration time across experimental phases S1, S2 and S3. **C**) Discrimination index indicating exploration of displaced versus stationary objects during S3. Boxplots show the median and interquartile range (B), and bars (C) represent the mean ± SEM. Individual data points are overlaid to illustrate the distribution within each group. Asterisks indicate statistical significance at *p < 0.05, **p < 0.01, ***p < 0.001. *N* = 8 per group. **D)** Recording of fEPSPs at Schaffer collateral-CA1 synapses before and during the item-place task (red circles: propranolol-treated mice, blue circles: vehicle-treated mice). Freely behaving animals received test-pulse stimulation during session exposure. Novel item-place exposure in S1 resulted in LTD in controls. Re-exposure to the same item-constellation in S2 failed to elicit a change in synaptic strength. Exposure to a new item-place configuration in S3 resulted in LTD. Propranolol treatment prevented this sequence of responses. Line breaks indicate change in time-scale. Analogs represent fEPSPs recorded during the individual experiments at time points that correspond to the numbering in the graph. Black arrow depicts the time of injection, or respective session commencement. Vertical scale bar: 2 mV, horizontal scale bar: 8 ms. *N* = 6 per group.

One day before novel item-place exposure, animals were habituated to the arena and baseline recordings of Ca^2+^ activity were obtained. On the test day, mice received an intraperitoneal (i.p.) injection of either vehicle (0.1 ml/g body weight), or the β-AR antagonist, propranolol (20 µg per gram of bodyweight at a volume of 0.1 ml/g). Thirty minutes later, the item-place task commenced, consisting of three sessions separated by 60-minute intervals comprising: **Session 1**: Novel item-place Exposure (10 min), in which animals explored two distinct novel objects that were inserted into the recording chamber; **Session 2**: item-place re-exposure (10 min), whereby animals were exposed to the same object configuration; and **Session 3**: item-place reconfiguration (10 min), in which one object’s position was changed relative where it had been in the 1st and 2nd sessions (Fig. 1A).

In vehicle-treated animals, the time spent exploring objects varied significantly across sessions (Repeated-measures ANOVA; F_2,23_ = 24.21, *p* < 0.001), with a statistically significant decrease evident during session 2 (*p* < 0.001) and session 3 (*p* < 0.01), compared to session 1 (Fig. 1B). During session 3, discrimination indices^41^ were calculated to determine how well mice distinguished between the displaced and stationary objects (Fig. 1C). Vehicle-treated mice showed a statistically significant preference for the displaced object (*p* = 0.003, one-Sample t-test against 0), consistent with the acquisition, stabilization and updating of item-place memory^41^. Analyses of locomotion activity (total distance traveled, average speed, total immobility bouts) revealed no significant differences between groups (Supplementary Fig. S1).

In propranolol-treated animals, object exploration times also differed across sessions (Repeated-measures ANOVA; F_2,23_ = 15.07, *p* = 0.001), but pairwise comparisons revealed no changes between session 1 and session 2 (*p* > 0.05), with a decrease in exploration time observed only within session 3 compared to session 2 (*p* < 0.05). Between group comparisons showed significant differences between groups (Repeated-measures ANOVA; F_2,28_ = 15.26, *p* < 0.001), with Tukey’s post hoc tests indicating that vehicle-treated mice spent more time exploring objects than propranolol-treated mice during session 1 (*p* < 0.01), while propranolol-treated mice spent more time exploring objects during session 2 (*p* < 0.05). Moreover, propranolol-treated mice had significantly lower discrimination indices than vehicle-treated mice during session 3 (independent samples t-test; t_(14)_ = 4.97, *p* < 0.001). The similar exploration times across session 1 and session 2, combined with the absence of a preference for the displaced item-place associations, indicate that propranolol-treated mice exhibited impairments in the encoding and updating of item-place associations.

### The acquisition and updating of item-place associations are associated with the emergence of long-term depression in CA1 that is hindered by β-adrenergic receptor antagonism

We previously showed, in a similar item-place learning task with 24-hour inter-session intervals, that both novel item-place exposure (S1) and item-place reconfiguration (S3) trigger β-AR-dependent long-term depression (LTD) in the murine dorsal CA1 region^6,41^. Here, we asked whether the current protocol, which used 1-hour inter-session intervals, likewise elicits CA1 LTD, and whether the single acute propranolol treatment used in this study is sufficient to block task-induced synaptic plasticity across sessions, or whether its effect wanes by the last session (Fig. 1D). Animals received test-pulse stimulation (5 pulses at 0.025 Hz, applied at 5-minute intervals) to evoked field excitatory post-synaptic potentials at Schaffer collateral- Stratum radiatum synapses of the dorsal CA1 region. They could move freely throughout the entire experiment. Exploration of two novel objects (session 1), during test-pulse stimulation, resulted in significant hippocampal LTD in vehicle-treated mice, compared to propranolol-treated mice that failed to exhibit LTD (Repeated-measures ANOVA: F1,10 = 27,805, *p* < 0.001). Re-exposure to the familiar objects (session 2) revealed no differences in evoked fEPSPs in vehicle- and propranolol-treated animals (Repeated-measures ANOVA: F1,10 = 0,51, *p* = 0.93). The former finding aligns with previous reports that LTD is not expressed when rodents are re-exposed to a now familiar environment^6,41^. The latter effect is consistent with retention of enough propranolol in the brain, such that LTD is impaired^6^. Displacement of one object (session 3) resulted in significant LTD in the control group, consistent with previous findings that spatial information updating promotes LTD^41^. Animals that received propranolol showed impoverished LTD (Repeated-measures ANOVA: F1,10 = 10,68, *p* < 0.01), although some recovery of synaptic depression was evident in the last 20 min of recordings, where evoked responses in control and propranolol-treated animals had become equivalent (Fisher LSD post hoc test: *p* = 0.24). These results show that in this paradigm, both acquisition and updating of item-place associations elicit β-AR-dependent LTD in CA1, and that β-AR antagonism disrupts learning-facilitation of synaptic plasticity for ca. 3 h after intraperitoneal (i.p.) injection.

### β-adrenergic receptors modulate the recruitment and reactivation of neuronal populations during item-place learning

**Fig. 2:**
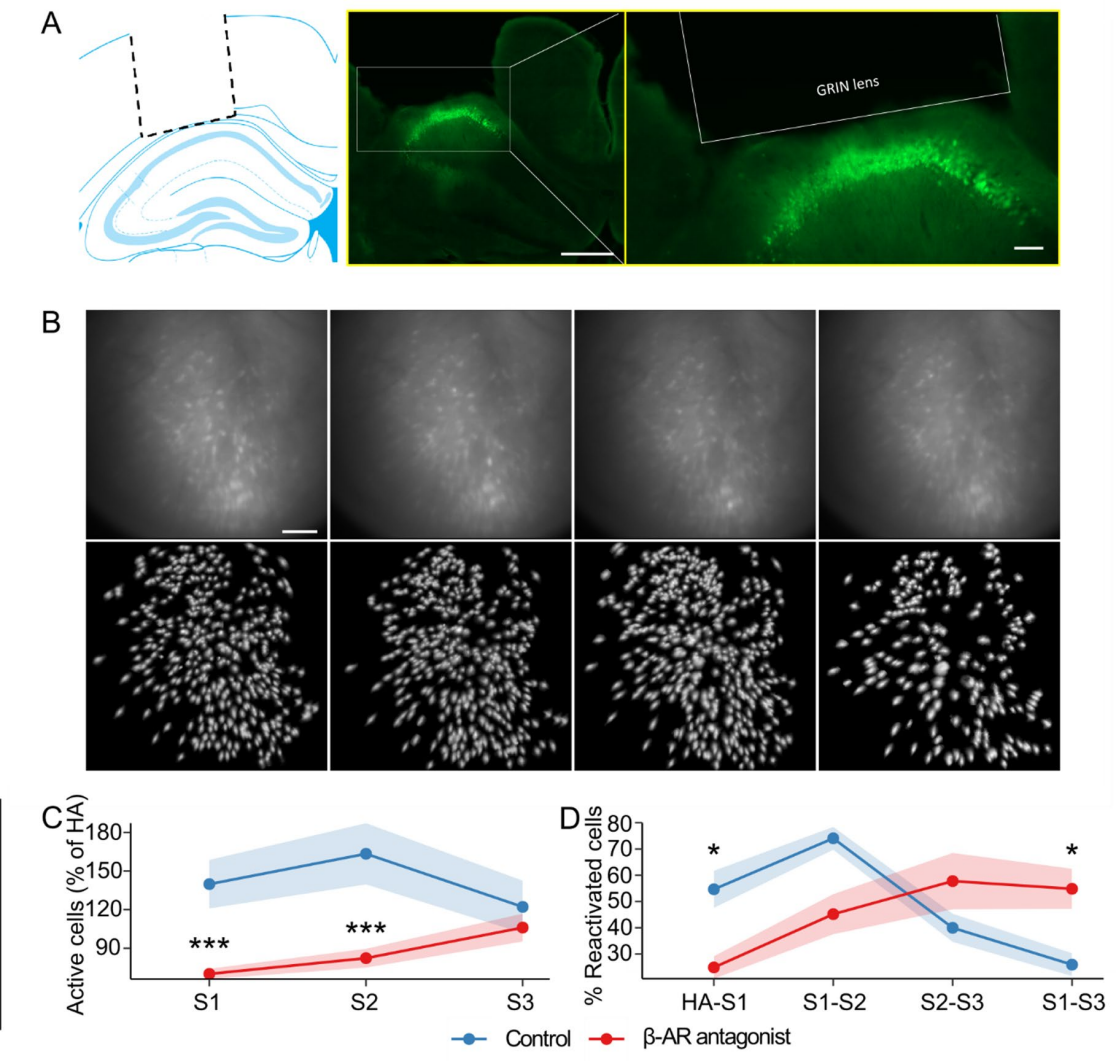
Recruitment and reactivation of neuronal populations during cumulative item-place learning. **A**) Left: Location of the GRIN lens over the dorsal CA1 (schema adapted from^42^. The dotted line shows the position of the lens over the pyramidal cell layer. Middle: white rectangle outlines pyramidal cells of the CA1 region of one mouse, that express GCaMP7f (green fluorescent label). Scale bar represents 500 μm. Right: Expansion of the same image shown in the middle panel indicating the grin lens location over the GCaMP7f-labeled cells. **B**) Top row: Raw field of view through the miniscope showing CA1 pyramidal cells detected in each session of one mouse. Left to right: habituation (HA), novel exposure (session 1, **S1**), re-exposure (session 2, **S2**) novel item configuration (session 3, **S3**). Scale bar represents 100 μm. Bottom row: maximum intensity projection of cell maps corresponding to the upper panels. **C**) Percentage of active cells normalized to habituation (HA) baseline values across item-place sessions S1, S2 and S3. **D**) Percentage of cells that were reactivated from one item-place session to the next, as well as between habituation (HA) and S1. Points and shaded ribbons represent mean ± SEM. Asterisks indicate statistical significance at *p < 0.05, **p < 0.01, ***p < 0.001. *N* = 8 per group.

To investigate how β-AR influence neuronal dynamics during spatial learning, we performed wide-field Ca^2+^-imaging of the dorsal CA1 (Fig. 2A) using a miniature integrated microscope (miniscope) and microendoscopic GRIN lens^22^. This approach allowed us to record the activity of hundreds of neurons in freely behaving mice, while they performed behavioral tasks (Fig. 2B).

We first examined the recruitment of active neurons in CA1 across the task sessions (Fig. 2C). The number of active cells per session was normalized to baseline habituation values and compared between groups. In the vehicle group, pairwise comparisons revealed no significant changes in the percentage of active cells across pairs of sessions (*p* > 0.05, Wilcoxon signed-rank test), with only a trend toward a reduced number of active cells between session 2 and session 3 (*p* = 0.08).

In contrast, propranolol-treated mice exhibited significantly less activated cells than vehicle-treated mice during sessions 1 and 2 (*p* < 0.001, Wilcoxon rank-sum test). However, no difference in the percentage of activated cells was detected between controls and propranolol-treated animals during session 3 (*p* > 0.05). Within-group comparisons revealed significant changes across sessions in the propranolol group (*p* = 0.01, Friedman rank-sum test). These findings indicate that the relative percentage of neuronal participation was stable in controls across sessions, but that propranolol treatment prior to session 1, not only reduced the percentage of actively participating neurons overall, but also altered the activation dynamics across sessions.

To clarify the extent to which the population of active neurons in sessions 1 through 3 was the same neurons or different ones, we then examined the percentage of cells that were recruited in one session that were activated again in subsequent sessions (Fig. 2D). Here, vehicle-treated mice displayed significant differences across session pairs (*p* < 0.01, Friedman rank-sum test). Specifically, they exhibited a sharp rise in neuronal reactivation of the same neurons between sessions 1 and 2, but showed a drop in reactivation of this population between sessions 2 and 3 (*p* < 0.01, Wilcoxon signed-rank test) and between sessions 1 and 3 (*p* < 0.001). This indicates that the reiteration of the item-place experience during session 2 recruited a similar population of neurons (that became active in session 1), but the change in spatial content in session 3, resulted in a change of ensemble properties.

The percentage of consistently reactivated cells was altered by propranolol treatment (Fig. 2D). Here, from session to session, the percentage of re-activated cells (relative to the entire population of activated cells) steadily increased (habituation vs. session1, session 1 vs. session 2, session 2 vs. session 3, *p* < 0.05).

In summary, vehicle-treated mice re-used previously active neuronal ensembles when contexts were similar, and did not preferentially recruit previous ensembles when a novel spatial content change was introduced. Propranolol-treated mice, failed to show stability in neuronal reactivation between session 1 and 2 and showed different neuronal reactivation patterns compared to controls during session 3.

### β-adrenergic receptor antagonism alters place cell-like properties

**Fig. 3:**
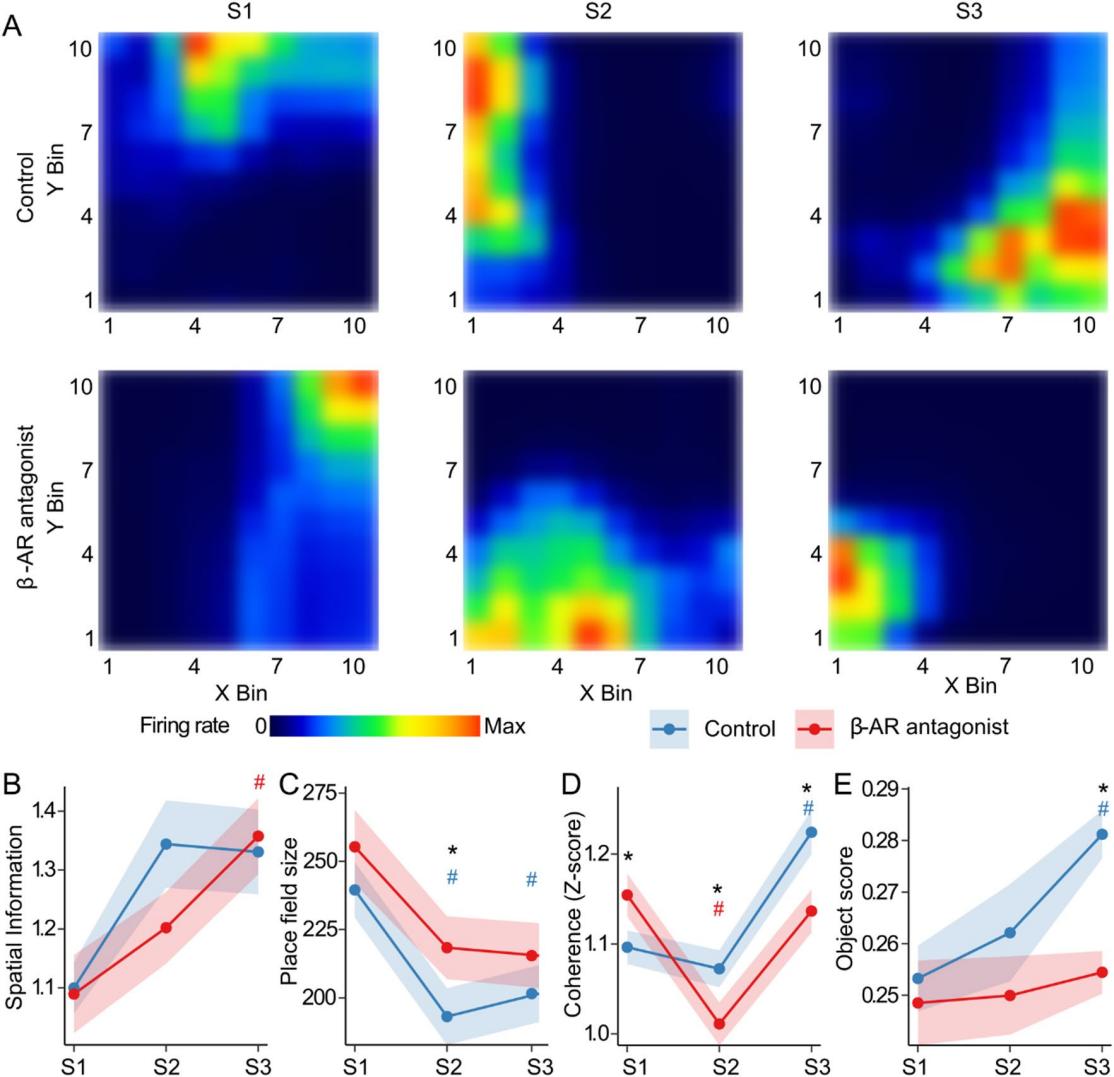
β-AR antagonism alters place cell-like properties. **A)** Representative firing rate maps of place-cell-like cells from each group and session of the item-place learning task: **S1** (novel item-place exposure), **S2** (re-exposure), **S3** (novel item configuration). Each map depicts a different cell. **B**) Spatial Information scores (bits/event). **C**) Place field sizes (cm^2^).

Despite the slower temporal dynamics of Ca^2+^ transients, compared to electrophysiological recordings from neurons, wide-field Ca^2+^-imaging enables the detection of spatially responsive cells using GCAMP7 sensors^43^- referred to here as place cell-like cells. To investigate CA1 spatial information processing in cumulative item-place learning and the role of β-AR in this process, we identified place cell-like cells (Fig. 3A) and quantified the following parameters: spatial information (bits/event), to assess how well cell activity encoded spatial locations; place field size, to determine the granularity of spatial representations; spatial coherence, to evaluate the local consistency of activity patterns; and object score, which reflects the percentage of place cell-like cells encoding locations near objects^44,45^.

In control animals, spatial information remained stable across sessions (*p* = 0.051) (Fig. 3B), whereas place field size showed a significant reduction (*p* < 0.001) (Fig. 3C), with pairwise comparisons revealing a decrease from session 1 to both session 2 and session 3 (*p* < 0.05). Spatial coherence also changed significantly across sessions (*p* < 0.0001) (Fig. 3D), remaining stable from session 1 to session 2 (*p* > 0.05), but increasing during session 3 (*p* < 0.0001). The proportion of place cell-like cells tuned to objects changed across sessions (*p* < 0.05), with a significant increase from session 1 to session 3 (*p* < 0.001) (Fig. 3E), suggesting *de novo* integration of information about the changed item configuration. These results indicate that, in control mice, place cell-like activity in CA1 becomes more spatially refined and coherent with repeated experience, and dynamically adapts to subtle changes in environmental features, such as changes in spatial content. This behavior is consistent with what one would expect of electrophysiologically recorded place fields, as well as their response to spatial content change within a known environment^46,47^.

β-AR antagonism resulted in different place cell-like properties across sessions. Spatial information content increased from session 1 to session 3 (*p* < 0.05) (Fig. 3B), and place field size decreased from session 1 to session 2, and session 1 to session 3 (*p* < 0.05) (Fig. 3C). The decrease in place field size was less pronounced in propranolol-treated animals than in controls during session 2 (*p* < 0.05). Spatial coherence followed a distinct trajectory (*p* < 0.0001) (Fig. 3D), with a sharp drop from session 1 to session 2 (*p* < 0.0001), which then returned to the level of session 1 during session 3 (*p* > 0.05), but was still lower than controls (*p* < 0.01). Unlike controls, the object scores did not change across sessions (*p* = 0.8) and were significantly lower than those of controls during session 3 (*p* < 0.0001) (Fig. 3E).

Together, these findings suggest that during item-place learning, place cell-like activity, detected via wide-field Ca^2+^ imaging in controls, becomes increasingly spatially resolved and coherent with experience, and dynamically adjusts to changes in item location. By contrast, β-AR antagonism disrupts this dynamic, particularly by impairing spatial coherence and spatial tuning to the objects, potentially reflecting impaired encoding of novel spatial features/content of the environment.

**D**) Z-scored coherence measure. **E**) Object scores showing average overlap of place fields with object’s vicinity. Points and shaded ribbons represent mean ± SEM. Asterisks indicate significant differences between groups at *p < 0.05. Hashtags indicate significant within-group differences at ^#^*p* < 0.05 from S1, with blue denoting the control group and red denoting the β-AR antagonist group. *N* = 6 per group.

### β-adrenergic antagonism dampens population bursts in CA1

**Fig. 4:**
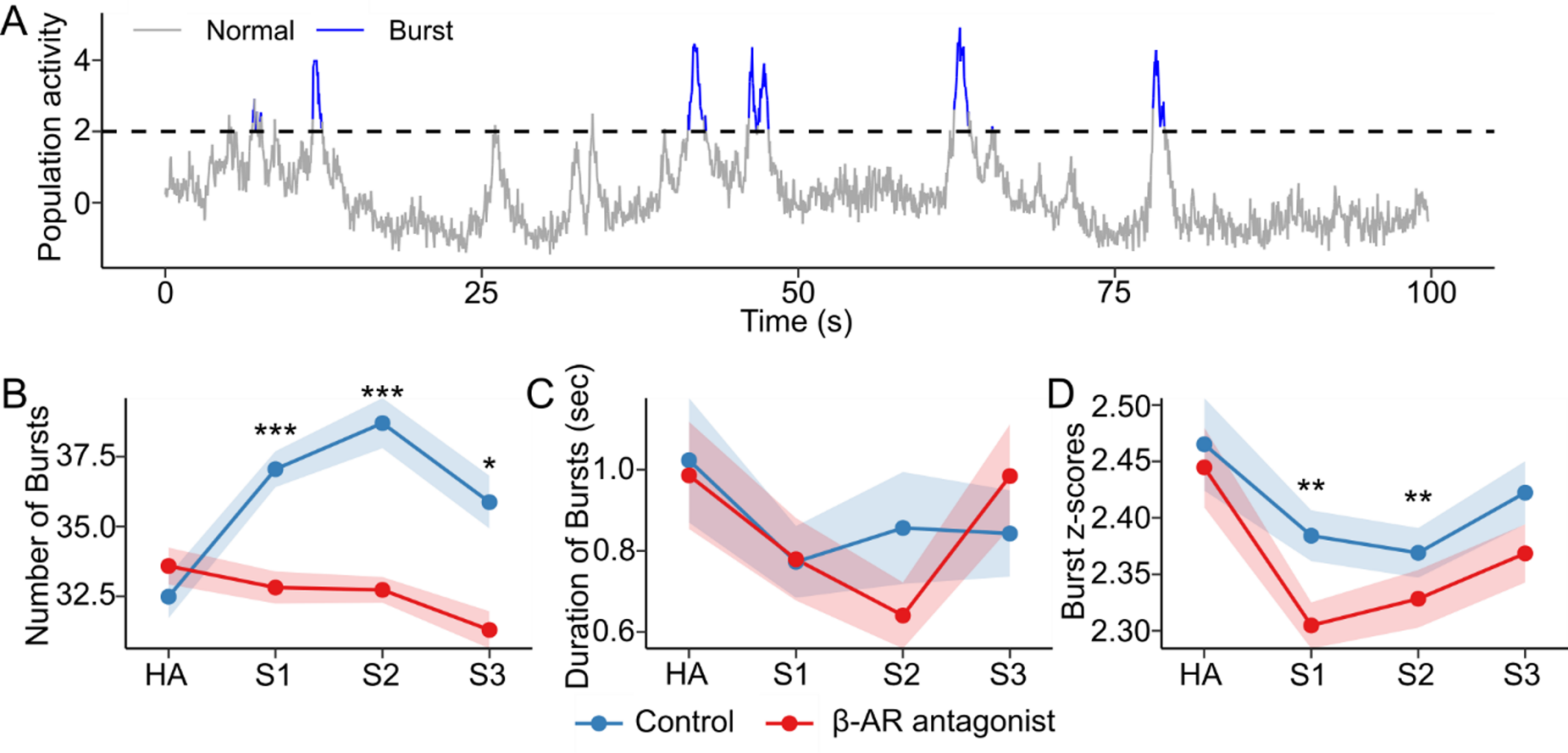
Population bursts in CA1. **A**) Z-scored mean population activity in a representative segment of CA1 recordings. The dashed line indicates the threshold (z = 2) for identifying burst events (highlighted in blue). **B**) Average number of burst events per group across sessions including habituation (HA), S1 (novel item-place exposure), S2 (re-exposure), S3 (novel item configuration). **C**) Average duration of burst events (in seconds) per group across sessions. **D**) Average z-scores of burst events per group across sessions. Points and shaded ribbons represent mean ± SEM. Asterisks indicate statistical significance at *p < 0.05, **p < 0.01, ***p < 0.001. *N* = 8 per group.

Hippocampal function is characterized by population bursts - brief events during which large groups of neurons co-activate. These burst events support memory consolidation^18,48^. These events were initially described electrophysiologically, but are detectable to some extent through Ca^2+^-imaging, despite the slower temporal resolution of neuronal Ca^2+^ sensors^16,18,49^. In our recordings, CA1 neurons displayed periodic bursts of synchronized activity (Fig. 4A). To quantify these bursts, we analyzed their number, duration, and magnitude across sessions and compared these metrics between groups.

In the vehicle group, the number of population bursts differed across sessions (*p* < 0.001, Kruskal-Wallis test) and this number was elevated, compared to habituation, during both sessions 1 and 2 (*p* < 0.001, Wilcoxon signed-rank test) (Fig. 4B). Moreover, a slight increase was observed from session 1 to session 2 (*p* = 0.04). Both the duration and magnitude of bursts were stable across sessions (*p* > 0.05, Kruskal-Wallis test). As previously reported in linear track appetitive tasks^43^, but unlike in fear-conditioning paradigms^18^, CA1 population activity was correlated with the animal’s speed (Supplementary Fig. S2 A-B). However, this correlation was less pronounced during burst events (Supplementary Fig. S2 C-D) and did not differ between groups or across sessions. Burst events coincided with brief increases in velocity relative to 5 s before or after the burst peak, but not relative to 2.5 s (Supplementary Fig. S2 E). The proportion of neurons participating in burst activity was stable across sessions and groups and was not influenced by speed (Supplementary Fig. S2-F).

The antagonism of β-AR changed population burst activity. The number of bursts varied across sessions (*p* = 0.02, Kruskal-Wallis test) (Fig. 4B), with pair-wise comparisons revealing a decrease during session 3 compared to habituation (*p* < 0.001, Wilcoxon signed-rank test) and lower than controls during sessions 1 and 2 (*p* < 0.001, Mann-Whitney test) and session 3 (*p* < 0.05, Mann-Whitney test). Burst duration remained stable and did not differ between groups (*p* > 0.05) (Fig. 4C). However, burst magnitude, indicating the intensity of synchronized neuronal activity^50^, changed significantly across sessions (*p* < 0.001, Kruskal-Wallis test) and was reduced during sessions 1 and 2 compared to habituation (*p* < 0.01, Wilcoxon signed-rank test), and significantly lower compared to controls (*p* < 0.01, Mann-Whitney test) (Fig. 4D). In both groups, the majority of the bursts lasted for no longer than a couple of seconds. We detected a small fraction lasting longer than 5 s (Vehicle group: 3.1%; Propranolol group: 2.8%), which likely represent trains of consecutive bursts that – due to the slower temporal resolution of calcium dynamics – were detected as a single prolonged burst.

These findings indicate that β-AR regulate neuronal firing properties. Given the role of hippocampal bursts in supporting memory consolidation^18^, this disruption suggests that propranolol impairs CA1-mediated item-place memory consolidation by dampening synchronous neuronal activity.

### β-adrenergic receptor signaling influences network connectivity in CA1

**Fig. 5:**
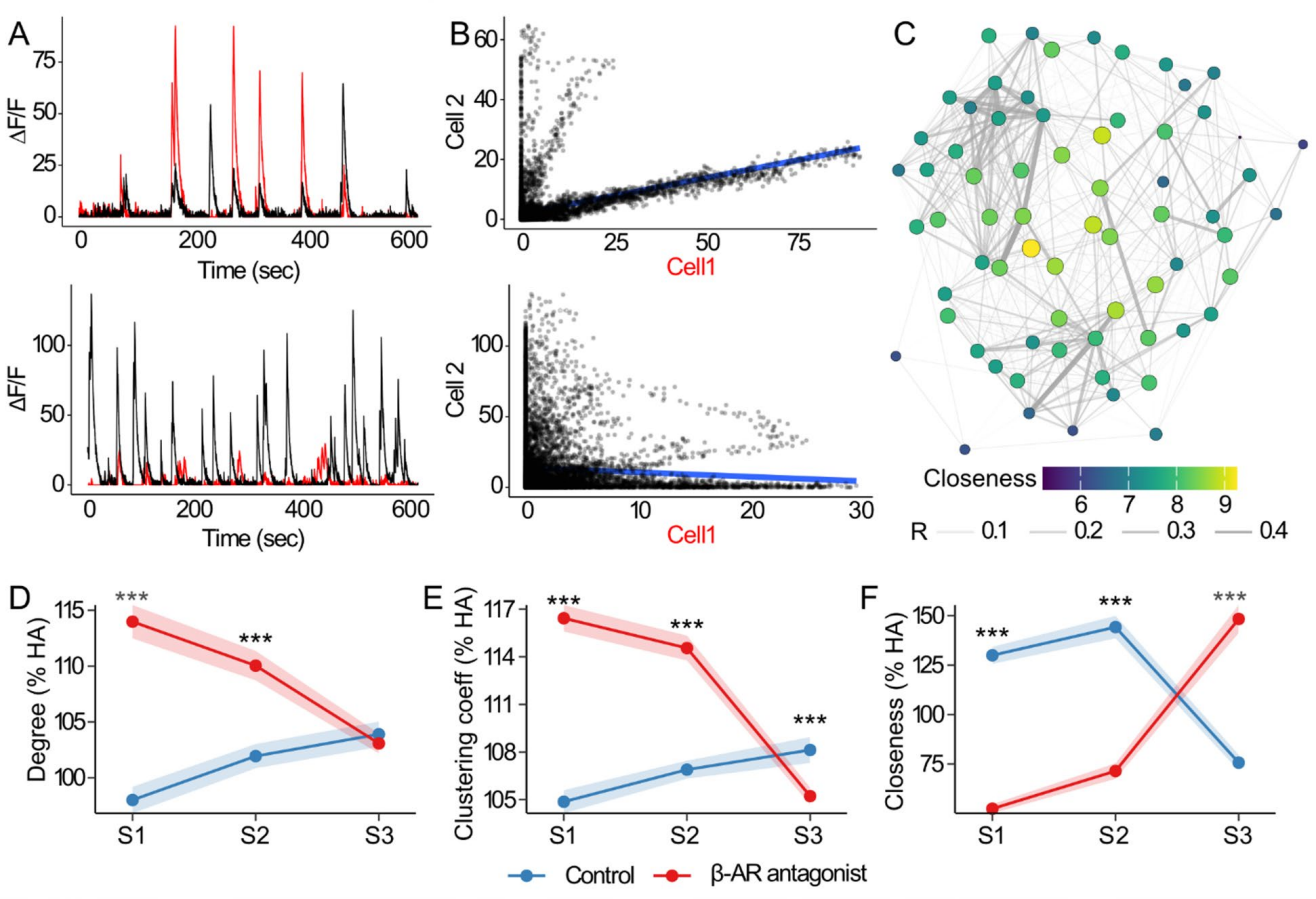
Functional network connectivity in dorsal CA1. **A**) Example of overlaid Ca^2+^ signals (∆F/F) from pairs of cells (cell 1 in red lines, cell 2 in black lines) that were co-active (top), or not (bottom). **B**) Example of correlations between pairs of cells that were significantly correlated (top) or not (bottom). Dark dots represent individual Ca^2+^ signals paired by time, and blue line represents the line of best fit. **C**) Example of a functional network of CA1 cells generated after thresholding for positive correlations (*P* < 0.001). Edges represent correlation coefficient values, and node size and color indicate closeness centrality. **D**) Degree centrality values, indicating synchronously active cells, expressed as a percentage of baseline values from the habituation session. **E**) Clustering coefficient values, indicating the proportion of mutually interconnected cells, expressed as a percentage of baseline values from the habituation session. **F**) Closeness centrality values, indicating the accessibility of the nodes in the network, expressed as a percentage of baseline values from the habituation session. **D-F)** S1 (novel item-place exposure), S2 (re-exposure), S3 (novel item configuration). Points and shaded ribbons represent mean ± 95% confidence intervals. The control group is colored in blue, and the β-AR antagonist group is colored in red. Asterisks indicate statistical significance at *p < 0.05, **p < 0.01, ***p < 0.001. *N* = 8 per group.

To understand how neuronal interactions in CA1 evolve during spatial learning and how they are affected by β-AR blockade, we performed network analyses^51^ based on cellular Ca^2+^ transients detected during the item-place learning sessions. Functional connectivity networks were constructed by calculating Spearman correlations of ∆F/F signals between all cell pairs in each session (Fig. 5A-B). Significant positive correlations were retained to generate functional connectivity networks (Fig. 5C), and centrality measures were calculated to capture patterns of information processing. Centrality values from subsequent sessions were normalized to the habituation (HA) baseline.

In control networks, ‘degree’ (Fig. 5D), reflecting the number of synchronously active cells, changed across sessions (*p* < 0.001, Kruskal-Wallis test) and showed a progressive rise (*p* < 0.05, Wilcoxon Rank Sum test). The clustering coefficient (Fig. 5E), reflecting the proportion of cells connected to nodes that are also interconnected, also increased (*p* < 0.001, Kruskal-Wallis test), with significant rises occurring from session 1 to session 2, and session 1 to session 3 (*p* < 0.001). The clustering coefficient remained stable, however, between sessions 2 and 3 (*p* > 0.05). Closeness centrality (Fig. 5F), reflecting how easily a node can reach all others, was stable from session 1 to session 2 (*p* > 0.05), but dropped sharply from session 1 to session 3 (*p* < 0.001). This drop, along with the increased degree and clustering, suggests a shift towards network compartmentalization in control animals, that may be necessary to integrate novel information.

The functional connectivity of CA1 networks was profoundly changed by β-AR antagonism. In contrast to responses in the control group, degree values decreased progressively across sessions (Fig. 5D) (*p* < 0.05, Wilcoxon Rank Sum test). Degree was significantly higher in propranolol-treated networks compared to control networks during sessions 1 and 2 (*p* < 0.001, Mann-Whitney test), with no difference occurring during session 3 (*p* > 0.05).

Moreover, in contrast to vehicle-treated animals, clustering in propranolol-treated networks declined progressively across sessions (*p* < 0.001) (Fig. 5E), and, while it was significantly higher than controls during session 1 and 2 (*p* < 0.001), it became lower during session 3 (*p* < 0.001).

Closeness values also diverged from vehicle-treated controls under propranolol treatment (Fig. 5F). Closeness increased progressively across sessions (*p* < 0.001) and while it was lower than in controls during sessions 1 and 2 (*p* < 0.001) it became significantly higher during session 3 (*p* < 0.001). This progressive increase in closeness across sessions, despite decreasing degree and clustering, suggests a less specialized, more uniform network state that fails to reorganize effectively in response to novel information.

In summary, vehicle-treated networks transitioned from a sparse, efficient configuration (high closeness, low degree and clustering) to a denser, modular state (lower closeness, higher degree and clustering), in sessions 1 through 3, supporting the integration of new information, while maintaining previously encoded content. Following propranolol-treatment, networks diverged from this pattern, initially exhibiting high degree and clustering but low closeness, indicating redundancy and inefficient information flow. Over time, decreasing degree and clustering with rising closeness suggest a reliance on global connectivity without the modular organization needed to integrate spatial information effectively.

## Discussion

A fundamental challenge in neuroscience is understanding how hippocampal ensembles dynamically process information during spatial learning. The dorsal CA1 region is central to these processes^13,48,52^, with noradrenergic inputs playing a key modulatory role^7,38^. Although dorsal CA1 neuronal activation patterns support memory, mediated by the storage, recall, and updating of spatial experience, the specific role of β-AR in regulating related neuronal population activity remains poorly defined. Here, we employed a spatial object recognition paradigm known to enable hippocampal plasticity that depends on β-AR^6^ to investigate, by means of Ca^2+^-imaging, cellular dynamics during spatial learning and memory updating. We show that cumulative item-place learning and its updating not only enable hippocampal LTD, but are also associated with the progressive recruitment and reactivation of CA1 ensembles, refinement of spatially tuned neurons, consolidation-linked population bursts, and circuit connectivity changes to support the integration of new information. In a separate group of mice, the β-AR antagonist, propranolol, was administered acutely (i.p.) prior to the first learning session (S1) to disrupt β-AR-mediated signaling during initial information encoding. This experimental design allowed us to examine how an early perturbation of β-AR-dependent information encoding influences later memory processes that are dependent on the initial learning event. By tracking cellular dynamics across sessions, we further sought to assess whether hippocampal activity patterns gradually recover, or reorganize following acute β-AR antagonism. We confirmed that β-AR are required for the manifestation of LTD during spatial information acquisition and updating and, moreover, show that activation of β-AR are needed for the establishment of neuronal ensembles that support these spatial learning events.

In the item-place learning task, animals were exposed to the same context across three sessions: first encountering two objects (session 1), then re-encountering them in the same arrangement (session 2), and finally encountering a configuration where one object was displaced (session 3; Fig. 1A). This design allows animals to encode, recall/re-iterate, and update spatial representations and thus, provides a basis for the examination of dynamic, cumulative spatial learning. Successful learning is indicated by a reduction in exploration time from session 1 to session 2, reflecting the successful acquisition of item-place memory. Memory recall and subsequent updating is indicated by the increased exploration of the displaced object during session 3. Consistent with previous work on the importance of β-AR in spatial learning and consolidation^5,53,54^, and particularly in associating objects with locations^6^, animals treated with the β-AR antagonist, propranolol, failed to exhibit these behavioral indicators of learning. They also failed to express LTD.

Reactivation of cellular ensembles active during learning underlies memory recall^9,10^, while encoding new information and updating memories relies on integrating novel ensembles^4^. To be able to distinguish between memory acquisition, reactivation/reiteration of the memory and memory updating, we separated our sessions by 60 min. Animals explored in each session for 10 min. This duration of novel spatial exploration has been shown in the past to be an adequate time-span for the manifestation of place fields^45^, and of learning-facilitated synaptic plasticity^30^, whereas an interval of at least one hour before re-exposure to the same spatial environment results in stable place fields^55^. Moreover, spatial learning triggers nuclear immediate early gene (IEG) expression in the CA1 region, whereby some of the neurons that were activated by the first spatial experience are recruited and/or modified by subsequent novel item-place learning^28^. We hypothesized that NA acting via β-AR facilitates item-place associations by supporting information processing in CA1 ensembles at different functional levels. Accordingly, we expected that acute pharmacological antagonism of β-AR signaling during initial spatial information encoding would weaken task-related ensemble recruitment and reactivation, impair the refinement of spatial tuning, and alter population-level burst activity and connectivity patterns across sessions.

The intervals between sessions were relatively short (60 min). Thus, we first assessed whether the session events resulted in the same profile of learning-facilitated hippocampal synaptic plasticity, as was demonstrated in the past for mice (using 24 h intervals between events) or rats (using 7 day intervals between events)^30^. We observed that consistent with previous reports, initial exposure of the mice to a novel spatial item constellation, during test-pulse stimulation of Schaffer collaterals, resulted in LTD of Stratum radiatum synapses of the dorsal CA1 region^41^. Re-exposure to the same item configuration 1 h later failed to facilitate LTD, whereas exposure to the familiar items in an unfamiliar spatial constellation a further 1 h later, resulted in *de novo* LTD. These findings align with the abovementioned reports that rodents express hippocampal LTD when exposed to novel spatial content, or an updating of spatial aspects of this content^30^, and also show that this phenomenon can occur even if sleep consolidation is largely absent between behavioral events.

Although we have already shown that antagonism of β-AR prevents learning-facilitated LTD under very similar learning conditions^6^, there is controversy in the peer-reviewed scientific literature as to the duration of efficacy of propranolol in the brain, when applied via different routes to rodents, with half-lives reported to be between one and several hours^79–81^. If propranolol was only effective in preventing responses during the first learning session and associated LTD assessment, one would expect to see learning and LTD during the second event (because learning had been impaired in the first session). If propranolol was still effective during the second session, we would not expect to see LTD (and learning) but these would emerge during session 3 if propranolol had been eliminated from the brain by then. We observed that LTD was significantly impaired during the 3rd session, although some recovery of LTD was evident towards the end of the one-hour recording period, indicating that after i.p treatment of mice with propranolol, its efficacy at brain β-AR persists for ca. 3 h.

To explore whether information storage and updating related to item-place learning was represented by large populations of CA1 neurons and whether the detected neuronal ensembles change in an experience-dependent manner, we used the cell activity recorded during the habituation session as a reference, and then determined how many of these cells were active in session 1. We detected a distinct population of CA1 neurons that responded to this first learning event. We observed that a large percentage of the same cells were also active in session 2, when the item-place information was repeated, suggesting re-engagement of an established engram ensemble to support recall. This property of hippocampal neurons has been demonstrated using optogenetics in studies of fear memory recall^56^ and using chemogenetic tagging of neuronal ensembles^57^.

Reactivation of the cells that were active during sessions 1 and 2 sharply declined in controls when the spatial content (item-pace configuration) was changed during session 3 (session 2 vs. session 3 and session 1 vs. session 3), indicating a lesser engagement of previously activated ensembles when the new item-place configuration was being learned. This finding is consistent with the updating of spatial content information in CA1 neuronal ensembles^28^. In fact, re-activation of the original session 1 ensemble in its entirety might be expected to disrupt context-dependent information updating by preventing new ensemble connectivity from occurring^36^.

These interpretations are supported by our observations in animals that were treated, prior to session 1, with a β-AR antagonist and failed to exhibit item-place learning, consistent with previous reports^41^. These mice exhibited reduced ensemble reactivation, compared to controls, during the novel spatial experience of session 1 and no significant change in activity from session 1 to session 2. Interestingly, and in contrast to controls, reactivation of the neuronal ensemble increased from session 2 to 3 (and between session 1 and 3).

Antagonist treatment was given 30 min before commencing session 1, and 170 min elapsed between the timepoint of injection and the beginning of session 3. Although we did not measure the propranolol concentration in brain tissue, our LTD findings suggest that it was still efficacious at antagonising β-AR in session 3. No differences in evoked responses were evident between controls and propranolol-treated animals was evident at the end of the recording session, however, indicating that propranolol efficacy had started to decline during session 3. Also, neuronal recruitment in session 3 reached similar levels to that in controls, and at the behavioral level, exploration in session 3 was also similar to that of session 2 in controls, which suggests a decreasing effect of propranolol that became relevant ca. 3 h after i.p. administration.

Taken together, this pattern of neuronal activity observed during β-adrenergic receptor antagonism may indicate a reduced ability of the hippocampus to discriminate between novel and familiar information. One parsimonious interpretation is that, during session 3, mice relied relatively more on *de novo* information encoding rather than on the updating of representations acquired during sessions 1 and 2. These findings are consistent with reports that activation of β-AR is needed for stabilization of recently acquired spatial memory^58^. Thus, our findings suggest that the activation of β-AR may support the re-use of previously acquired ensembles during recall and facilitate the recruitment of new ensembles when spatial configurations changes.

Although Ca^2+^-sensors do not have the temporal resolution of electrophysiological recordings, it has nonetheless been demonstrated that spatially responsive cells can be discriminated with wide-field Ca^2+^-imaging^43^. To examine the extent to which the detected neuronal ensemble activity could align with place cell activity in the dorsal CA1 region, we examined spatial information (bits/event), place field size, spatial coherence, and object score^44,45^. In control animals, we observed that place cell-like cells became more spatially refined and coherent in session 3 compared to session 1, consistent with the stabilization of place fields over time and familiarity with the spatial environment^55,59^. Spatial coherence increased in session 3 compared to the other sessions and object scores increased. Additional analysis showed that among all place-cell metrics, only the object score showed a significant positive correlation with the discrimination index in session 3 (Supplementary Fig. S3). Taken together with the abovementioned finding that fewer members of the original neuronal ensemble were re-activated in session 3, this finding is consistent with refined cellular dynamics that update hippocampal representations to reflect changes in allocentric information content^46,47^ at temporal intervals that require information retrieval and updating^60^. To further validate these interpretations, we analyzed population bursts of neurons in the three sessions. These bursts, characterized by periodic, highly synchronous cellular activity, occur in CA1^3^ and contribute to memory consolidation and updating^17,18,61^. Bursts can be detected using wide-field Ca^2+^ -imaging, despite the slower temporal resolution of neuronal Ca^2+^ sensors^16,18,49^. We observed periodic bursts of synchronized activity in the CA1 that increased in number from session 1 through 2, but decreased in session 3, whilst remaining significant from burst activity recorded during habituation. Although bursts have been primarily associated with memory encoding^8^, they were also elevated during S2 when recall was tested. We speculate that this may reflect the temporal proximity between sessions (1 h), coinciding with a time point in which offline encoding-related burst activity has been reported^18^. Alternatively, additional spatial or contextual information may have been encoded during S2, given the animals’ sustained arena exploration despite reduced object exploration (Supplementary Fig. S1). Burst firing is also a characteristic of place cells^60^. It is tempting to speculate that this change in activity during session 3 may have been driven by endogenous LTD that occurs in the CA1 region during novel item-place learning, as well as information updating^41^, and is known to modulate place fields^62^.

During spatial object learning and updating, β-AR antagonism disrupted place cell-like stability and decreased spatial coherence. Findings are consistent with reports by others that locus coeruleus inputs to the hippocampus regulate place fields in a state-dependent manner^40^. β-AR antagonism also reduced the number of population bursts detected across sessions. These findings suggest that β-AR modulate burst activity in CA1, as reported for NA action in hippocampal slices^63,64^ and the antagonist-mediated disruption of burst firing may have contributing to the observed memory deficits at the behavioral level^65^. Furthermore, the altered burst dynamics likely contribute to the changes in cellular recruitment and reactivation observed across sessions.

Because brain function depends on the coordinated activity of sparse populations of neurons^8^, we also examined how β-AR antagonism influences CA1 circuit-level interaction. This was done by constructing functional connectivity networks^51,66,67^. In control animals, the network evolved from a sparse, efficient configuration in session 1 (low degree and clustering, high closeness) to a denser, more modular architecture during session 3 (high degree and clustering, lower closeness), indicating compartmentalization for integrating new information. In contrast, antagonist-treated networks were dense in session 1, albeit inefficient at distributing information and ultimately became sparser, with information transfer efficiency peaking late in session 3 of the item-place task. This inverted trajectory likely hindered their ability to effectively encode novel inputs and recall previous information, further underscoring the role of β-AR in supporting CA1 network reconfigurations to accommodate changing demands.

Given the absence of group differences in mean Ca^2+^-event frequency or amplitude, the behavioral and ensemble-level effects of propranolol are more consistent with β-AR-dependent modulation of synaptic plasticity than with global changes in excitability. In line with this, electrophysiological recordings using the same experimental design showed that propranolol significantly prevented task-induced LTD in CA1 (Fig. 1D). This indicates that β-AR signaling supports the updating and stabilization of item–place representations by enabling plasticity within CA1 circuits, which in turn shapes ensemble recruitment, spatial tuning, population burst and network connectivity across sessions.

In summary, our findings reveal that proper spatial learning emerges from coordinated patterns of cellular recruitment, spatial representation, synaptic plasticity and population-level dynamics within dorsal CA1. Importantly, antagonism of β-AR disrupts cumulative spatial learning and information updating, inhibits hippocampal synaptic plasticity in the form of LTD, and alters related CA1 hippocampal ensembles at several levels. Our use of systemic propranolol treatment raises the possibility that some of these effects may stem from broader network influences, rather than direct antagonism of β-AR within the CA1 region, and future research should aim to disentangle these influences and identify key modulatory regions. Collectively, these results show that to encode and update spatial memories CA1 ensembles undergo multiscale dynamic changes, from cellular recruitment to changes in synaptic strength and network reorganization, and that β-AR are essential modulators of this process.

## Methods

### Animals

The study was conducted in accordance with the European Communities Council Directive for care of laboratory animals (2010/63/EU) and ARRIVE guidelines (https://arriveguidelines.org). The experiments were approved in advance by the local state authority (Landesamt für Verbraucherschutz und Ernährung, North Rhein-Westfalia). All efforts were made to minimize the number of animals used for this study, specifically by conducting power calculations to establish the minimal cohort size for meaningful statistical analyses.

Experiments were performed on eight male CBA/CaOlaHsd mice (9–11 weeks at the time of surgery; Envigo, Germany or in-house breeding). This mouse strain does not exhibit sensory deficits thoughout its lifespan, and exhibits superior spatial learning behavior and hippocampal synaptic plasticity compared to mouse strains that have chronic sensory impairments e.g. C57Bl/6 mice^68,69^. Mice were housed in sibling groups in a temperature and humidity-controlled vivaria (Scantainer Ventilated Cabinets, Scanbur A/S, Denmark) with constant 12-hour light-dark cycle (lights on from 7 a.m. to 7 p.m.), controlled temperature (22 ± 2 °C), humidity (55 ± 5%). Food and water were available ad libitum throughout all experiments. After the surgery mice were housed individually and experienced at least 7 days of recovery before the commencement of experiments.

Female mice were not included because they become stressed by the presence of male mice^70^ and this could alter the outcome of the behavioral experiments. For this reason, we used males only (reduction of variability of responses and keeping animal numbers low without loss of statistical power). At the end of study, animals were euthanized by placing them in a sealed chamber pre-saturated with isoflurane (Isofluran CP, 1 ml/ml solution, CP-Pharma Handelsges. mbH, Burgdorf, Germany). Rapid loss of consciousness was followed by respiratory arrest. Death was confirmed by cessation of heartbeat.

### Surgical procedures

Surgical procedures were adapted from methods established by others^22^. At 9–11 weeks of age, mice were anesthetized with sodium pentobarbital (60 mg/kg, intraperitoneally (i.p.)) and were head-fixed in a stereotaxic frame. After scalp hair removal, local analgesia was applied using a 10% Lidocaine spray (Xylocain^®^ pump spray, Aspen Germany GmbH, München, Germany).

Pre- and postoperative analgesia was implemented via an s.c. injection of Meloxicam (Metacam, 0.2 mg/kg, Boehringer Ingelheim, Ingelheim am Rhein, Germany), immediately prior to and 24 h after surgery.

After leveling the skull (i.e. to a horizontal plane of 0^o^) along the anterior-posterior (AP) axis of the skull, the same correction leveling was conducted for the medio-lateral (ML) axis, at a distance of 2 mm from the center of the line (describing the fusion of skull-bone plates) separating bregma and lambda. The depth of anesthesia was monitored during the whole surgical procedure by checking that the tail or foot pinch reflex was absent^71^.

### Ca^2+^ sensor treatment

Following a craniotomy (0.5 mm in diameter), 500 nL of GCaMP7f-expressing Adeno-Associated Virus solution (PGP-AAV-syn-jGCaMP7f-WPRE, 1.8 × 10¹³ vg/mL, Catalogue no. 104488-AAV1, Addgene), diluted with sterile 0.9% saline solution, was injected through a glass micropipette (Science Products GmbH, Germany), targeting dorsal CA1 (AP: −2.1, ML: −1.4, DV: −1.3 mm with respect to bregma). Fifteen minutes after the injection, the pipette was slowly removed. The cavity was filled with bone wax (Sharpoint^®^, Surgical Specialties, Tijuana, Baja California, Mexico). The scalp was sutured and povidone-iodine solution (10%, Betaisodona^®^, Mundipharma GmbH, Frankfurt am Main, Germany) was applied over the sutured area. Ten days after this procedure, Gradient Refractive Index (GRIN) lens (part# 1050–004605, Inscopix Inc., Palo Alto, California, USA) implantation was conducted. Animals received anesthesia and analgesia as described above. The site for lens implantation was marked on the skull at AP: −2.2, ML: −2.1 mm. To limit the drilling area to the diameter of the GRIN lens, 3 locations around the craniotomy mark were additionally marked at a distance of 0.5 mm in the anterior, posterior and lateral directions. Two additional holes were drilled, one anterior to and another posterior to the left coronal suture to anchor two stainless steel screws (1 mm, Helix, Villingen-Schwenningen, Germany). Following the craniotomy (1 mm in diameter), aspiration of the cortical tissue overlying the dorsal CA1 was carried out under constant supply of cold sterile physiological saline. Aspiration continued until the removal of medial-lateral striations of the corpus callosum, revealing the anterior-posterior striations. The GRIN lens (1 mm diameter, 4 mm length, product code: 1050–004623, Inscopix Inc., Palo Alto, California, USA) was implanted at a 9-degree angle over the CA1 (AP: −2.2, ML: −2.1 mm, DV: ~ −1.4 mm) under constant visual monitoring through the operation microscope (Model OPMI 1-FC, Carl Zeiss, Oberkochen, Germany). Once the GRIN lens was implanted, the physiological saline solution located in the gaps between the skull edge and the GRIN lens, was removed and replaced by cyanoacrylate adhesive (UHU^®^ Sekundenkleber, UHU GmbH & Co KG, Bühl, Germany). Acrylic dental cement (Paladur, Heraeus Kulzer GmbH, Hanau, Germany) was further applied onto the dried cyanoacrylate adhesive, and around the screws affixed to skull. Fourteen days after lens implantation mice were anesthetized with sodium pentobarbital (60 mg/kg, i.p.) and their GCaMP signal was confirmed using a miniscope (Inscopix Inc., Palo Alto, California, USA) set at the middle of its focal range and attached to a baseplate (product code: 1050–004638 Inscopix Inc., Palo Alto, California, USA). The miniscope was connected to an nVista 2.0 imaging system (Inscopix Inc., Palo Alto, California, USA). After acquiring an in-focus view of dorsal CA1 region, the baseplate was glued to the skull using dental cement (Paladur^®^ Heraeus Kulzer GmbH, Hanau, Germany). Parameters associated with miniscope imaging such as focus level, excitation light intensity, and gain were also finalized. At 3–4 days prior to the recording sessions, mice were handled by the experimenter and habituated for a minimum of 20 min, twice a day to the procedure of attaching the miniscope. This was carried out in the same experiment room where the imaging chamber was located, in order to habituate the mice to the experiment environment. The same experimenter handled all mice during all handling and experiment days.

### Pharmacological treatment

The competitive β-AR antagonist, propranolol hydrochloride (Tocris Bioscience, Abingdon OX14 3NB, United Kingdom), was dissolved in 0.9% NaCl solution and administered intraperitoneally (i.p.) at a dose of 20 mg/kg and at a volume of 0.01 ml/g, and applied 30 min before novel exposure session 1, as described previously^39^. Control animals were injected with vehicle solution (0.9% NaCl, 0.1 ml/g).

### Item-place learning task

We used a cumulative item-place task to examine the ability of the mice to distinguish between an object that is located at a familiar location or an object that is spatially novel^6^. The task took place in a 40 × 40 × 50 cm arena. Item-place experiments were performed in three 10 min sessions separated by 60 min intervals (Fig. 1A). On day 1, Ca^2+^ signals were recorded at 20 FPS while mice explored the arena in the absence of any physical items (habituation). On day 2, during session 1 (novel exploration), two novel objects were presented for exploration. The mice were then re-exposed to the objects in the same locations in the next session (session 2), and in the third session (session 3) one object was shifted, in a parallel fashion, to the other side of the arena to test for item-place recall and information updating. Objects as well as their relative positions were randomly assigned for each animal. The imaging chamber was cleaned thoroughly between task trials to ensure the absence of olfactory cues. After every trial and before the first presentation, the objects were cleaned with ethanol, rinsed with water and dried. The objects were distinctly different from one another and heavy enough so that they could not be moved by the mice. Several copies of each object were available.

Behavioral data for the item-place experiments were recorded from a camera (acA-1300, Basler AG, Ahrensburg, Germany) using EthoVision XT software (Noldus Information Technology, Wageningen, The Netherlands). Exploration of the objects was then analyzed post-hoc using the within-object area scoring system which was defined as sniffing of the object (with nose contact or head directed to the object) within ~ 2 cm radius of the object^72^. Standing, sitting or leaning on the object was not scored as object exploration.

### Statistical analysis of behavioral data

Behavioral data were analyzed using either repeated-measures ANOVA followed by Tukey’s post hoc tests or independent samples t-tests. Object exploration indexes were also compared against 0 using a one-sample t-test.

The Shapiro–Wilk test indicated that data related to cellular recruitment and reactivation, population bursts, place cell metrics, and network properties were often non-normally distributed. Therefore, non-parametric statistical tests were used for both within-group and between-group comparisons. Kruskal–Wallis tests were used to detect overall differences across sessions. When significant, these were followed by Wilcoxon rank-sum tests for within-group comparisons across sessions. Mann–Whitney U tests were used for pairwise between-group comparisons. The p-values were adjusted for multiple comparisons using the Holm method.

These analyses were conducted using R (version 4.2.2) using the R packages igraph (https://igraph.org/r), rstatix (https://CRAN.R-project.org/package=rstatix) and custom code written in R available at https://github.com/johaubrich.

### Signal and statistical analysis of wide-field imaging data

Extraction of Ca^2+^ signals from individual neurons was carried out using Inscopix Data Processing Software (IDPS) (Inscopix, Palo Alto, CA, USA). Preprocessing stages involved (i) selecting the region of interest (cropping), (ii) spatial down sampling by a factor of two, (iii) spatial filtering and (iv) motion correction. Putative cells were identified using ‘constrained non-negative matrix factorization for microendoscope data’ (CNMFe) toolbox^73^. The signal from an individual cell in each cell-set was then carefully checked through visual curation to make accept/reject decision, based on the location of signal relative to the cell contour and the corresponding appearance of the peak in the signal time trace. Only data from cells labelled as ‘accepted’ were used to create spatial footprints needed for alignment and longitudinal cell registration in the CellReg toolbox as described by Sheintuch and colleagues^74^. The habituation session was treated as the reference session, as it provides a scaffolding network over which further experiences during the item-place task were built. To compare active cells between the sessions, the cell count in each session was normalized to the habituation session count and expressed as percentage value. Reactivation of cells in a pair of successive sessions was also computed. For this, the percentage of cells in a given session that were reactivated in the subsequent session was calculated for each pair of sessions as shown in Fig. 2D (see example in Supplementary Fig. S4). Differences between groups were determined by Mann-Whitney U tests. Within-group comparisons were calculated using Friedman rank sum test followed by Wilcoxon signed-rank test.

Bursts were identified by first z-scoring the Ca^2+^ traces of all individual neurons recorded in a session and then computing the population mean activity at each time point. This population signal was subsequently z-scored, and periods exceeding a threshold of z = 2 were considered candidate bursts. To define a burst event, we marked the onset when the activity crossed above the threshold and continued to hold above it until it fell below the threshold again. Bursts shorter than 0.05 s were discarded to exclude transient fluctuations. Differences between groups were determined by Mann-Whitney U tests.

For functional network analysis, data containing changes in cellular fluorescence intensity were used. For each animal and session, pairwise Spearman correlations with Bonferroni correction between all cells were computed and only correlations with p values < 0.001 were considered as ‘edges’ in the resulting network. Different measurements of centrality were calculated to elucidate the connectivity patterns of the networks^60,75,76^. The degree of each node was calculated as its number of edges and was normalized by the overall network size. The clustering coefficient of each node estimated the likelihood of its neighbors being interconnected and incorporated the edge weights. Closeness centrality of each node was defined as the inverse of its average distance to all other nodes in the network, also taking into account the edge weights (r values). Differences between groups were determined by Mann-Whitney U tests. Within-group comparisons were calculated using Kruskal-Wallis test followed by Wilcoxon signed-rank test.

### Identification of place cell-like activity

Raw x-y positions, extracted from the tracking software, were converted from pixels to centimeters and down-sampled to a temporal resolution of 0.5 s. Denoised, deconvolved Ca^2+^ transients were treated as spike events. The arena was divided into 10 × 10 bins (~ 4 cm x 4 cm each) and each spike event was assigned based on its coordinates. Bins without events were filled with zeros, and the proportion of spatial bins visited (occupancy) was calculated.

Raw firing-rate maps were generated for every recorded neuron. Rate maps were denoised by convolving both the spike histogram and the occupancy histogram with an isotropic Gaussian kernel of width = 1. Spatial selectivity was measured with the Skaggs information metric as previously described^70^. Statistical significance was assessed by a circular-shuffling procedure: for each neuron the entire event train was rotated in time by a random offset while the behavioral record remained fixed, and the information score was recomputed 500 times. The empirical p-value or a neuron was the proportion of shuffled scores that equaled or exceeded the observed score; neurons with *P* < 0.05 were retained for further analysis. To ensure adequate sampling, neurons displaying fewer than 20 events during the recording were rejected. A cell was finally classified as a place cell-like cell only if it satisfied both criteria: significant spatial information (*P* < 0.05) and a minimum of 20 “spike” events.

The spatial extent of individual place fields was identified on each smoothed rate map by thresholding at 20% of the peak firing rate, labelling only contiguous above-threshold regions, as described by Harvey and colleagues^77^. For every accepted field the centroid coordinates and field size were recorded, and multi-field were allowed.

To calculate spatial coherence^44,45^, after Gaussian smoothing, the unsmoothed rate matrix and its box-car–smoothed counterpart were vectorized and their Pearson correlation coefficient was obtained over all spatial bins that contained at least one sample. A Fisher’s transform was applied, yielding a coherence score that increases with the spatial contiguity of firing.

To detect place-cell-like cells tuned to bins containing objects, a neighborhood extending two bins beyond each object’s centroid bin in every direction was defined, and a mask was created. To quantify how strongly a field overlapped an object region an “object score” was calculated. The thresholded smoothed rate maps were intersected with the padded object mask, and a ratio of overlap was calculated, ranging from 0 (no overlap) to 1 (the field entirely was contained within an object zone). Statistical analyses of place-cell properties were conducted at the cell level, rather than on per-animal averages. In two animals, the number of detected place cells across experimental sessions was deemed insufficient (Supplementary Fig. S5), and they were excluded from this analysis.

### In vivo electrophysiology approach and statistical analysis

For the assessment of synaptic plasticity in the item-place task, a total of twelve CBA/CAOlaHsd mice (in-house breeding) underwent chronic stereotaxic implantation of a bipolar stimulation electrode and a monopolar recording electrode based on previous studies^69^. All mice had a minimum weight of 22 g and an age of 8–10 weeks before undergoing surgical electrode implantation. They received meloxicam (Metacam, 0.2 mg/kg, Boehringer Ingelheim, Ingelheim am Rhein, Germany) as an analgesic before, as well as 24 and 48 h after surgery. Animals were anesthetized prior to surgery with sodium pentobarbital (60 mg/kg i.p.). The stimulation electrode was implanted at coordinates 2.0 mm anterior-posterior (AP), −2.0 mm mediolateral (ML) from bregma and ~ (-) 1.4 mm dorsoventral (DV) from brain surface corresponding to the Schaffer collateral (SC) pathway of the dorsal hippocampus. The recording electrode was implanted in the ipsilateral CA1 Stratum radiatum (AP: −1.9 mm, ML: 1.4 mm, DV ~ (-)1.2 mm) and was used to monitor the evoked potentials at SC-CA1 synapses during the implantation procedure. The coordinates used for the electrodes were based on the mouse brain atlas^78^. Test-pulse recordings performed during the surgery to determine the correct depth of the electrodes. The stimulation and recording electrodes were made of polyurethane-coated stainless-steel wire (100 μm diameter; Gündel, BioMedical Instruments, Zöllnitz, Germany) and were lowered into the brain through a single hole (~ 1.6 mm in diameter) that was drilled through the cranial bone. On the contralateral side, two additional holes (~ 0.7 mm in diameter) were drilled though the bone into which two anchor screws were inserted. Stainless steel wires (A-M Systems) were attached to the screws, serving as reference and grounding electrodes, respectively. The five wires were secured to a six-pin socket (Conrad Electronic SE, Hirschau, Germany) and the whole assembly was fixed on the skull using dental acrylic (J. Morita Europe GmbH, Dietzenbach, Germany; Haræus Kulzer GmbH, Dormagen, Germany). The animals were allowed at least 14 days for recovery before experiments were conducted. During this period, animals were monitored closely for infections or distress and were handled regularly. 24 h prior to start of the experiment, animals were transferred from their housing cages to the recording chamber with full access to food and water to ensure adequate familiarization of the environment.

The socket of each mouse was connected via a custom-made flexible cable that was attached to a swivel connector to the recording/stimulation system and allowed free movement of the mouse. Field excitatory postsynaptic potentials (fEPSPs), recorded in the dorsal CA1 region, were used to measure changes in synaptic responses which were generated in the Stratum radiatum by stimulation of SC at low frequency (0.025 Hz) with single biphasic square waves of 0.2 ms duration per half-wave, that were delivered by a constant current isolation unit (World Precision Instruments). The fEPSP signal was amplified using a differential AC amplifier (A-M Systems Science Products GmbH, Hofheim, Germany) and digitized through a data acquisition unit (Cambridge Electronic Design, Cambridge, UK). An input-output curve (stimulation intensity of 20, 30, 40, 50, 75, 100, 125 and 150 µA with a 5-minute interval) was determined immediately prior to commencing the experiment. Test-pulse stimulation in all experiments was conducted using the stimulation intensity that produced an fEPSP that was 40% of the maximum fEPSP obtained by means of the input-output curve. In the case that object exploration during session 1 resulted in synaptic depression in control experiments, the test-pulse stimulation intensity was increased prior to the next item exposure event (to re-attain the abovementioned 40% response). 

Six animals in the control group received vehicle (0.9% physiological NaCl) intraperitoneally (i.p.), the other six mice received propranolol (20 mg/kg at a volume of 0.01 ml/g, i.p.) 30 min prior to the first object exploration event. The item-place task was performed as described above.

For in vivo electrophysiology, each time point that was measured, consisted of the average of five consecutively evoked fEPSPs responses at 40 s intervals. The first six time points were recorded at 5-minute intervals and thus, served as baseline. The six time points were averaged and all time points throughout the experiment were expressed as the mean percentage ± standard error of the mean (SEM) of this value. The first three time points after each session were recorded with a five-minute interval and then data points were acquired every 15 min until 1 h had elapsed. Changes in synaptic transmission were determined by measuring the slope obtained on the first negative deflection of the evoked fEPSP. Between-group effects in electrophysiological data were assessed by means of a two-way ANOVA with repeated measures. A post hoc Fisher`s LSD test was used to discriminate significant effects at specific time-points/conditions. All statistical tests were performed using STATISTICA 13 (Statsoft, Tulsa, Oklahoma, USA). All tests were two-tailed, and significance was set at *p* < 0.05.

## Supplementary Information

Below is the link to the electronic supplementary material.

Supplementary Material 1

## Data Availability

The custom analysis codes and preprocessed data analyzed in this study are available on GitHub (github.com/johaubrich).
