# Supplementary material for: β-adrenergic receptors modulate CA1 population coding and synaptic plasticity during cumulative spatial memory formation and updating
Source: Sci Rep. 2026 Feb 19;16:7390. doi: 10.1038/s41598-026-40218-x (PMC12923906; doi:10.1038/s41598-026-40218-x)
Supplement: Supplementary file 1 — Supplementary Material 1 [file 41598_2026_40218_MOESM1_ESM.pdf]

Title: β-adrenergic receptors modulate CA1 population coding and synaptic plasticity during cumulative spatial memory formation and updating

Authors: Ninad Shendye, Josué Haubrich, Jens P Weber, Hardy Hagena, Denise Manahan-Vaughan

Ruhr-University Bochum, Medical Faculty, Department of Neurophysiology.

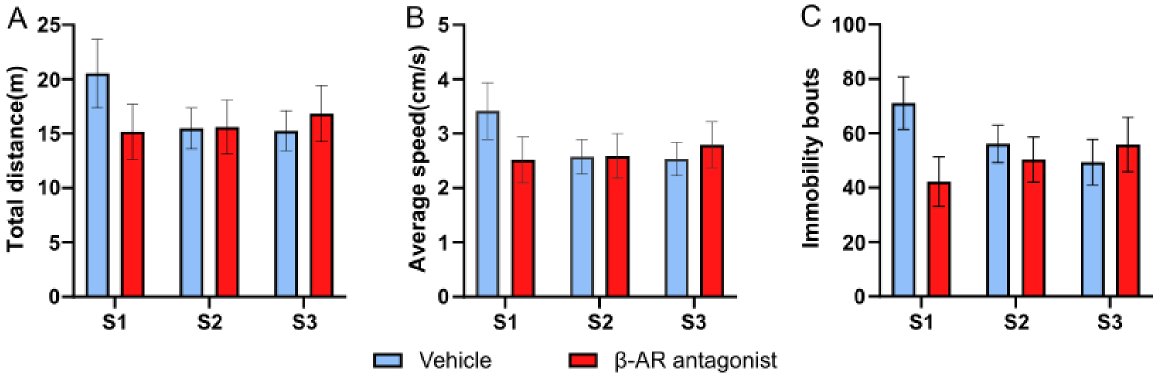

Supplementary Figure S1: Locomotion profile across item-place task

(A) Total distance travelled (meters) during each session of item-place task. No significant differences were detected between or within the groups (two-way ANOVA, $p > 0.05$).

(B) Comparison of average speed (cm/s) during the sessions showed no significant differences between or within the groups (two-way ANOVA, $p > 0.05$).

(C) Analysis of total immobility bouts showed no significant differences between or within the groups (two-way ANOVA, $p > 0.05$).

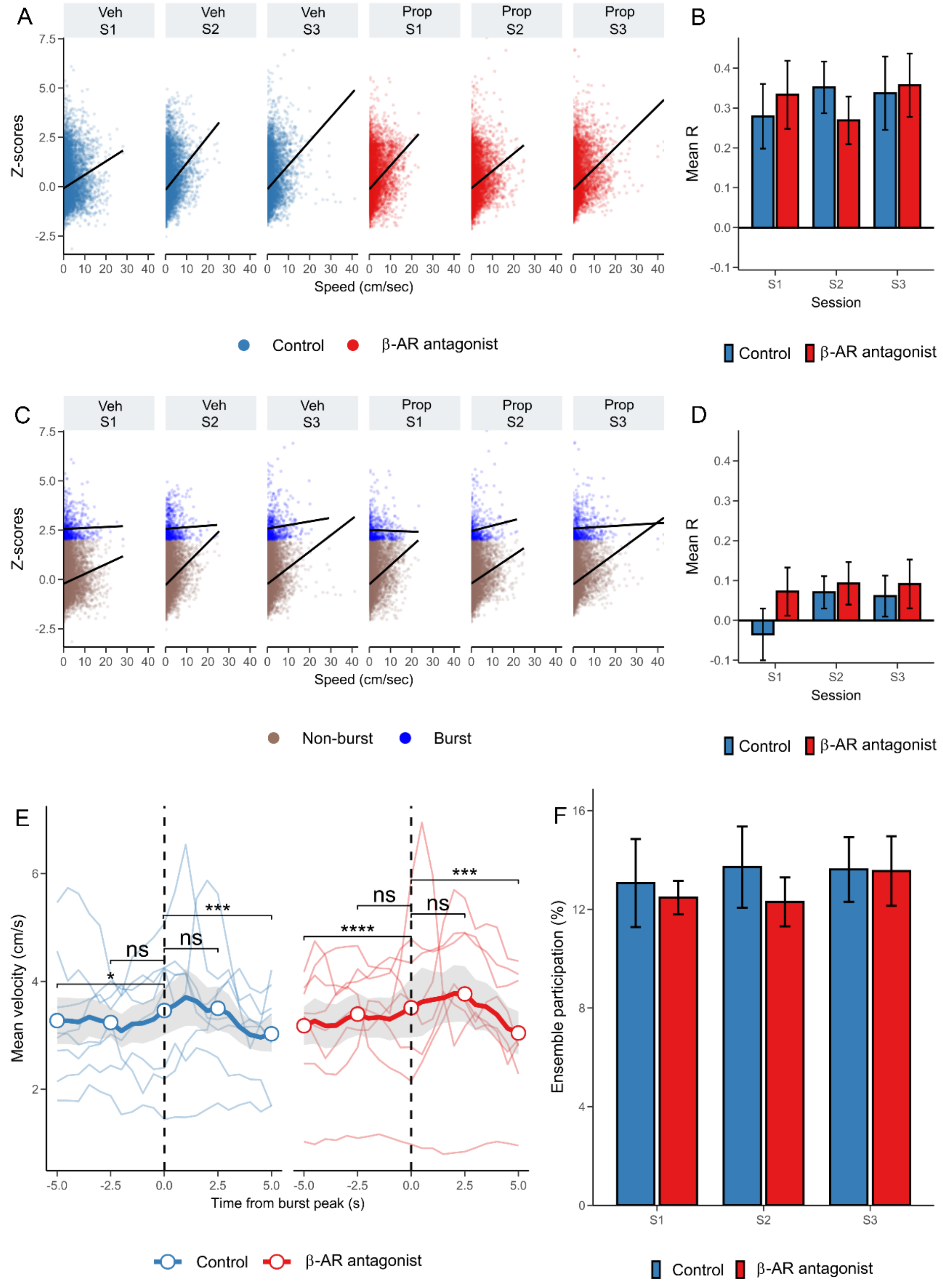

Supplementary Figure S2: Burst-related activity and locomotion.

(A) Correlation between z-scored population activity and speed (cm/s) across sessions in vehicle (left) and propranolol-treated mice (right). Population activity was significantly correlated with velocity in both groups across sessions.

(B) Comparison of mean correlation coefficients (R values) across sessions revealed no significant group differences.

(C) Correlation between z-scored population activity and velocity during burst events across sessions in control (left) and propranolol-treated mice (right). During bursts, velocity was weakly correlated with population activity in vehicle-treated mice during S3 ($R = 0.098$, $p = 0.017$) and in propranolol-treated mice during S2 ($R = 0.14$, $p < 0.001$), but not during other sessions ($p > 0.05$).

(D) Comparison of R values obtained during bursts showed no significant group differences.

(E) Average velocity surrounding burst peaks. Velocity traces were averaged across mice (thick line +- SEM ribbon), with thin lines indicating individual animals. Time was centered on burst peaks (0 s). Velocity during burst peaks was compared to 2.5 and 5 s before and after the peak. In both groups, velocity was higher at the burst peak compared to 5 s before or after (pairwise Wilcoxon tests with Benjamini–Hochberg correction, $p < 0.05$) but not compared to ±2.5 s ($p > 0.05$).

(F) Percentage of neurons participating in burst events. A neuron was considered active in a given burst if its z-scored activity exceeded 2. No significant group or session differences were observed.

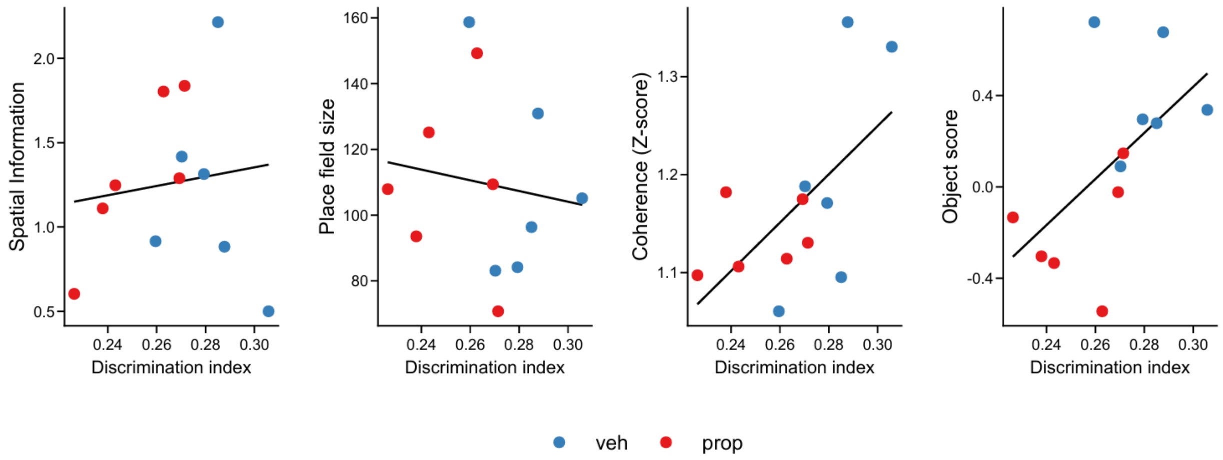

Supplementary Figure S3: Relationship between place-cell metrics and behavioral discrimination.

Correlation between the discrimination index during Session 3 (S3) and place-cell properties measured in the same session: (A) Spatial information, (B) Place-field size, (C) Spatial coherence, and (D) Object score. Among these measures, only the object score showed a significant positive correlation with the discrimination index ($R = 0.58$, $p < 0.05$).

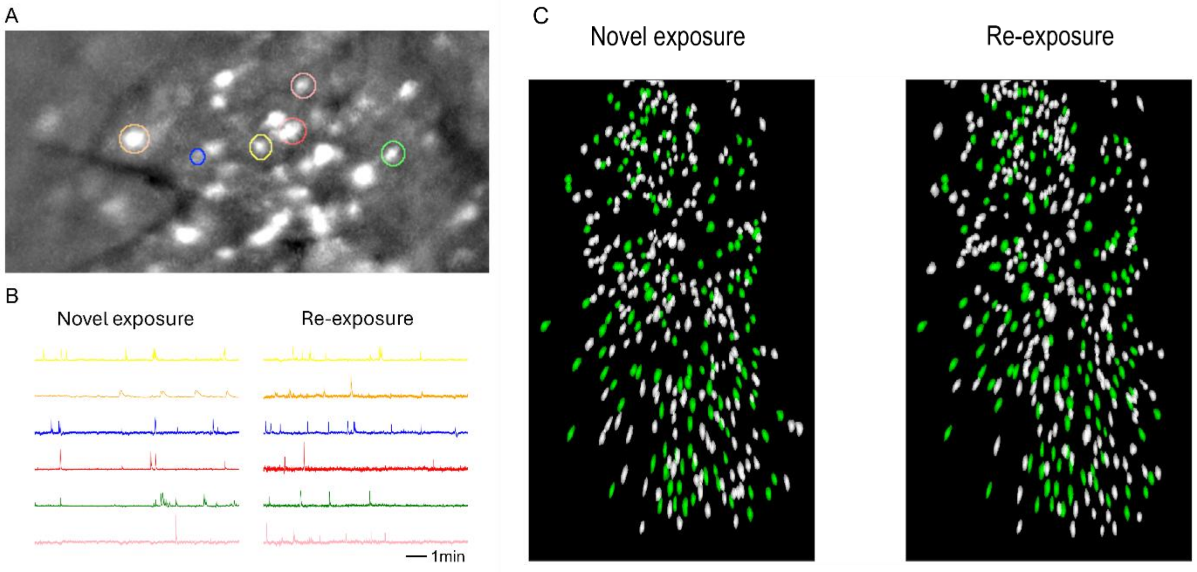

Supplementary Figure S4: Reactivation of the cells

(A) Contours show fluorescence activity from example cells that were detected in two subsequent sessions - novel exposure and re-exposure.

(B) Raster plots of detected neuronal activity of cells highlighted in A.

(C) Spatial footprints of cells detected in novel item-place exposure (session 1, left) and re-exposure (session 2, right) with cells reactivated in both sessions highlighted in green.

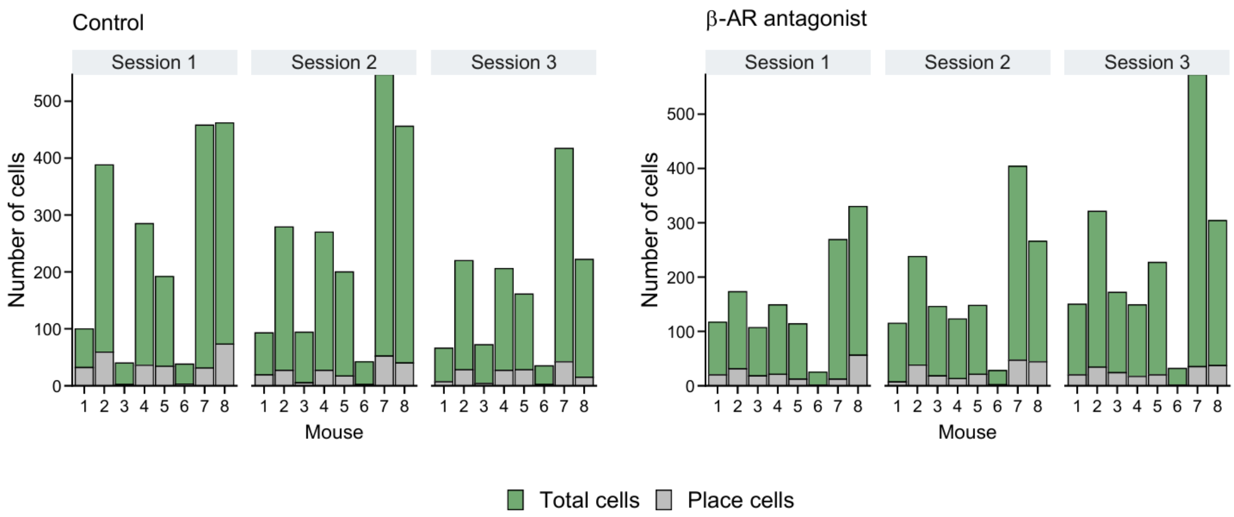

Supplementary Figure S5: Number of recorded and classified neurons.

Total number of neurons identified in each animal, session, and group, overlaid with the number of neurons classified as place cells. Two mice exhibited a very low number of detected place cells and were excluded from place-cell analyses, resulting in N = 6 for those analyses.
